# Nitric oxide induces apoptosis in a human colonic epithelial cell line, T84

**DOI:** 10.1155/S0962935195000391

**Published:** 1995-07

**Authors:** M. Sandoval, X. Liu, P. D. Oliver, X.-J. Zhang, D. A. Clark, M. J. S. Miller

**Affiliations:** 1 Department of Pediatrics Louisiana state University Medical Center New Orleans LA USA

## Abstract

Chronic inflammation is associated with inducible nitric oxide synthase expression in infiltrating and resident cells (epithelia, neurons) and an exaggerated release of nitric oxide. NO can induce apoptosis in macrophages and tumour cell lines. We investigated whether NO induced cell death in an epithelial (T84) cell fine via apoptosis. Culture T84 cells were exposed to a bolus of NO (40 or 80 μM) dissolved in Hank's balanced salt solution (HBSS) supplemented with 10% fetal calf serum (FCS). After incubation for 4 h at 37^°^C in 5% CO_2_, cells were either stained for DNA fragmentation with the TdT-mediated dUTP–biotin nick end labelling (TUNEL) method, or cytosolic DNA fragments quantified by a cell death detection ELISA assay. Nitric oxide induced apoptosis in a dose-dependent manner which preceded frank cell death (failure to exclude Trypan blue). These data suggest that epithelial cell death may be NO dependent and via apoptosis, in states of gut inflammation.


Mediators of Inflammation 4, 248–250 (1995)

### Nitric oxide induces apoptosis in a human colonic epithelial cell line, T84

M. Sandoval, X. Liu, P. D. Oliver, X.-J. Zhang, D. A. Clark, M. J. S. Miller[CA]

Department of Pediatrics, Louisiana State University Medical Center, New Orleans, LA, USA

[CA]Corresponding Author

**Chronic inflammation is associated with inducible nitric oxide synthase expression in infiltrating and resident cells (epithelia, neurons) and an exaggerated release of nitric oxide. NO can induce apoptosis in macrophages and tumour cell lines. We investigated whether NO induced cell death in an epithelial (T84) cell line via apoptosis. Culture T84 cells were exposed to a bolus of NO (40 or 80 μM) dissolved in Hank's balanced salt solution (HBSS) supplemented with 10% fetal calf serum (FCS). After incubation for 4 h at 37°C in 5% $CO_2$, cells were either stained for DNA fragmentation with the TdT-mediated dUTP–biotin nick end labelling (TUNEL) method, or cytosolic DNA fragments quantified by a cell death detection ELISA assay. Nitric oxide induced apoptosis in a dose-dependent manner which preceded frank cell death (failure to exclude Trypan blue). These data suggest that epithelial cell death may be NO dependent and via apoptosis, in states of gut inflammation.**

**Key words:** Apoptosis, Inflammation, Intestinal epithelial cell, Nitric oxide.

### Introduction

Most animal cells display a physiological form of cell death, which is influenced by the extracellular microenvironment. The mode of this cell death is often associated with characteristic morphological, biochemical and molecular changes.[1] Cells undergoing apoptosis demonstrate typical nuclear and cytoplasmic condensation followed by cell fragmentation. From the molecular standpoint, apoptosis is associated with the activation of nucleases that degrade the chromosomal DNA into oligonucleosomal fragments 180 – 200 bp in length.[2] While apoptosis is historically not linked to states of inflammation, it has become increasingly apparent that inflammatory mediators, e.g., oxidants and cytokines, can induce apoptosis.[3,4] Nitric oxide is a major secretory product of mammalian cells, with critical functions in homeostasis and host defence.[5] Chronic gut inflammation is associated with enhanced production of NO while inhibition of NOS ameliorates gut inflammation.[6,7] NO has been demonstrated to induce apoptosis in macrophages and tumour cells.[8,9] As the epithelium is a site of iNOS expression and marked cell death in gut inflammation,[10,11] we hypothesized that NO may induce apoptosis in cultured epithelial cells.

### Materials and Methods

*Cell culture:* T84 cells were obtained from the American Type Culture Collection (Rockville, MD, USA) at passage 52 on receipt. Cells were grown in a 1:1 mixture of Ham's F-12 medium and Dulbecco's modified Eagle's (high glucose) medium supplemented with 15 mM Na-*N*-2-hydroxyethylpiperazine-*N*-2-ethanesulfonic acid buffer (pH 7.4), 1 mM L-glutamine, 40 μg/ml penicillin, 90 μg/ml streptomycin, and 5% FCS. Medium $NaHCO_3$ concentration was 1.2 g/l, and cell cultures were maintained in a humidified 5% $CO_2$ incubator at 37°C. Harvested cells were plated in 6-well tissue culture plates and allowed to grow to confluence over 24 h before use.

*Preparation of NO solution:* NO solution was prepared as described previously.[12] Briefly, in a chemical hood, HBSS (GIBCO BRL, Gaithersburg, MD, USA) supplemented with 10% FCS was deoxygenated with 100% $N_2$ for 30 min. Subsequently, a 10% NO–90% $N_2$ gas mixture was bubbled into the flask for at least 30 min. The final concentration of this stock solution of NO was 160 μM, as previously determined by chemiluminescence.[12] Media pH was not altered by this procedure.

*Cell viability:* Aliquots of cells from control and NO-treated samples were examined for viability

© 1995 Rapid Science Publishers as determined by Trypan blue dye exclusion (0.04% Trypan blue dye in isotonic saline).[13] The number of non-viable cells was determined by light microscopy by counting those cells that failed to exclude the dye. Cells from each fraction were counted in a randomized manner using a haemocytometer.

*Detection of DNA fragmentation:* T84 cells were treated with either 0, 40 or 80 μM of dissolved NO and incubated for 4 h at 37°C in 5% $CO_2$. After incubation, cells were centrifuged at 200 × **g** for 5 min and a set of cells was used for DNA fragmentation assay using a cell death detection ELISA (Boehringer Mannheim, Indianapolis, IN, USA). Another set of T84 cells were stained for ***in situ*** apoptosis detection using the TUNEL (ApopTag detection kit, Oncor, Gaithersburg, MD, USA).

*Statistical analysis:* All data for the cell death detection ELISA is expressed as the mean ± S.E.M. Groups were compared by one-way analysis of variance. Analysis between groups was performed using the Tukey–Kramer multiple comparisons test.

### Results and Discussion

*Detection of DNA fragmentation:* Comparison of the cleavage of DNA into oligonucleosomal fragments revealed that DNA fragmentation increased in a dose-dependent manner in response to NO in the culture medium (Fig. 1). These results were validated with the ***in situ*** apoptosis detection kit (TUNEL). Cell viability by Trypan blue dye exclusion was negative for cells exposed to NO at concentrations up to 80 μM, indicating that DNA fragmentation preceded cell death. In other experiments, we have observed that cells exposed to concentrations of NO of 120 μM and higher did not survive the 4 h incubation period of this protocol, based on the failure of these cells to exclude Trypan blue and/or their detachment from culture dish. NO has been shown to induce apoptosis in macrophages and tumor cells.[8,9] In chronic gut inflammation NO synthesis is augmented via iNOS expression in both experimental animals and humans.[6,10,14] Our data indicate that, during gut inflammation, the overproduction of NO by iNOS in epithelia and infiltrating leukocytes may result in epithelial cell death via apoptosis.

Exaggerated NO synthesis in enterocytes of an inflammed mucosa may be regarded as a mechanism to establish a chemical barrier which is not conducive for translocation by intestinal

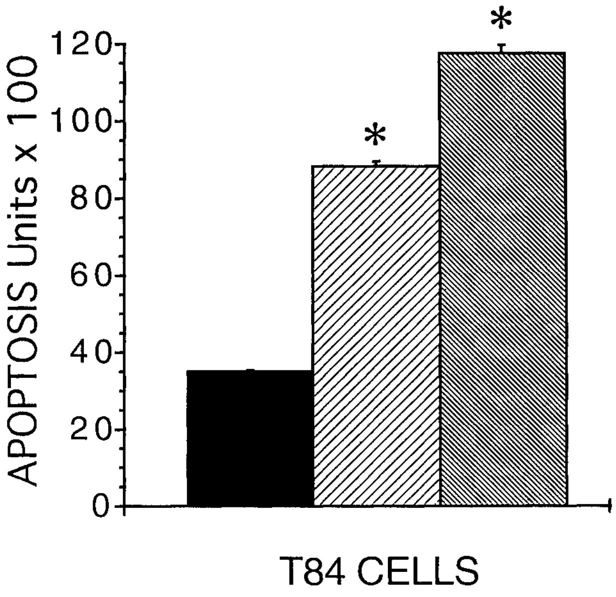

FIG. 1. Enrichment of oligonucleosomes in the cytoplasm of T84 cells treated with nitric oxide. DNA fragmentation in T84 cells treated with either 0, 40 or 80 μM NO. After NO treatment, cells were incubated for 4 h at 37°C in 5% $CO_2$ before homogenization, extraction of cytosolic DNA and analysis by cell death ELISA. ■, Control; ▨, 40 μM NO; ■, 80 μM NO.

flora. In so doing, the enterocyte is at risk of DNA damage. Apoptosis may be a defence against the survival of transformed cells. These effects of nitric oxide on epithelial cell death were observed with a dose of nitric oxide which must be considered transient, as it is oxidized within minutes under normoxic conditions to nitrite/nitrate, which alone were ineffective on cell death. The cascade of mechanisms initiated by this brief exposure to nitric oxide which culminates in apoptosis, remains to be investigated. Inflammatory bowel disease is a predisposing risk factor for colon cancer and we speculate that this may result from a failure to initiate programmed cell death in cells transformed by oxidant stress, excess nitric oxide or related species.

### References

1. Steller H. Mechanisms and genes of cellular suicide. *Science* 1995; **267:** 1445–1449.
2. Gerschenson LE, Rotello RJ. Apoptosis: a different type of cell death. *FASEB J* 1992; **6:** 2450–2455.
3. Sarafian TA, Bredesen DE. Is apoptosis mediated by reactive oxygen species? *Free Rad Res* 1994; **21:** 1–8.
4. Mekori YA, Oh CK, Metcalfe DD. IL-3 dependent murine mast cells undergo apoptosis on removal of IL-3. *J Immunol* 1993; **151:** 3775–3784.
5. Nathan C. Nitric oxide as a secretory product of mammalian cells. *FASEB J* 1992; **6:** 3051–3064.
6. Boughton-Smith NK, Evans SM, Hawkey CJ, *et al.* Nitric oxide synthase activity in ulcerative colitis and Crohn's disease. *Lancet* 1993; **342:** 338–340.
7. Miller MJS, Sadowska-Krowicka H, Chotinaruemol S, Kakkis JL, Clark DA.

Amelioration of chronic ileitis by nitric oxide synthase inhibition. *J Pharmacol Exp Ther* 1992; **264:** 11–16.

8. Albina JE, Cui S, Mateo RB, Reichner JS. Nitric oxide-mediated apoptosis in murine peritoneal macrophages. *J Immunol* 1993; **150:** 5080–5085.
9. Cui S, Reichner JS, Mateo RB, Albina JE. Activated murine macrophages induce apoptosis in tumor cells through nitric oxide-dependent or -independent mechanisms. *Cancer Res* 1994; **54:** 2462–2467.
10. Seago ND, Thompson JH, Zhang X-J, *et al.* Inducible nitric oxide synthase and guinea-pig ileitis induced by adjuvant. *Mediators Inflam* 1995; **4:** 19–24.
11. Tepperman BL, Brown JF, Whittle JR. Nitric oxide synthase induction and intestinal epithelial cell viability in rats. *Am J Physiol* 1992; **265:** G214–G218.
12. Iwamoto J, Krasney JA, Morin III FC. Methemoglobin production by nitric oxide in fresh sheep blood. *Respiration Physiol* 1994; **96:** 273–283.
13. Walker MW, Kinter MT, Roberts RJ, *et al.* Nitric oxide-induced cytotoxicity: involvement of cellular resistance to oxidative stress and the role of glutathione in protection. *Pediatric Res* 1995; **37:** 41–49.
14. Lundberg LON, Hellstrom PM, Lundberg JM, Alving K. Greatly increased luminal nitric oxide in ulcerative colitis. *Lancet* 1994; **344:** 1673–1674.

**Received 6 April 1995;**
**accepted in revised form 18 April 1995**
